# Time interval analysis of ductus venosus and cardiac cycles in relation with umbilical artery pH at birth in fetal growth restriction

**DOI:** 10.1186/s12884-021-04115-7

**Published:** 2021-10-03

**Authors:** Tomoki Suekane, Daisuke Tachibana, Yasushi Kurihara, Natsuko Yokoi, Naomi Seo, Kohei Kitada, Mie Tahara, Akihiro Hamuro, Takuya Misugi, Akemi Nakano, Masayasu Koyama

**Affiliations:** 1 Department of Obstetrics and Gynecology, Osaka City University Graduate School of Medicine, 1-4-3 Asahimachi Abeno-ku Osaka, Osaka, 545-8585 Japan; 2 Department of Obstetrics and Gynecology, Osaka City General Hospital, Osaka, Japan

**Keywords:** Ductus venosus, Cardiac cycle, Time interval, Fetal growth restriction, Umbilical artery pH

## Abstract

**Background:**

The aims of this study were to evaluate the time intervals of flow velocity waveforms (FVW) of ductus venosus (DV) and cardiac cycles, as well as the pulsatility index of DV-FVW (DV-PI), in correlation with umbilical artery (UA) pH at birth in fetal growth restriction (FGR) complicated with placental insufficiency.

**Methods:**

Data were retrospectively retrieved from pregnancies complicated by FGR. FGR was defined as an estimated fetal weight below − 2.0 S.D. with an elevated UA-PI. Time interval assessments of DV-FVW were as follows: the duration of systolic wave was divided by the duration of diastolic wave and defined as DV-S/D. We also measured the following time intervals of ventricular inflow through tricuspid valve (TV) and mitral valve (MV): (iii), from the second peak of ventricular inflow caused by atrial contraction (A-wave) to the opening of atrio-ventricular valves and: (iv), from the opening of atrio-ventricular valves to the peak of A-wave. (iii)/(iv) was expressed as TV-S/D and MV-S/D, for TV and MV, respectively. The time interval data were transformed into z-scores.

**Results:**

Thirty-one FGR fetuses were included in this study. Both DV-PI and DV-S/D showed significant correlation with UA-pH (*r* = − 0.677, *p* = < 0.001 and *r* = 0.489, *p* = 0.005 for DV-PI and z-score of DV-S/D, respectively) and more significances were observed in FGR ≤ 28 + 6 gestational weeks (*r* = − 0.819, *p* < 0.001 and *r* = 0.726, *p* = 0.005, for DV-PI and z-score of DV-S/D, respectively) than in FGR > 28 + 6 gestational weeks (*r* = − 0.634, *p* = 0.007 and *r* = 0.635, *p* = 0.020, for DV-PI and z-score of DV-S/D, respectively). On the other hand, TV-S/D and MV-S/D showed no significant correlation with UA-pH, although these z-scores indicated significant decreases compared with normal references.

**Conclusions:**

Time interval analysis of DV-FVW might be a valuable parameter, as well as DV-PI, for the antenatal prediction of fetal acidemia in the management of FGR fetuses.

**Supplementary Information:**

The online version contains supplementary material available at 10.1186/s12884-021-04115-7.

## Background

Fetal growth restriction (FGR) remains as one of the most challenging diseases in perinatal medicine, and close monitoring of fetal well-being is required to decide the optimal timing of delivery, especially for early-onset FGR fetuses complicated with placental insufficiency [1–4]. Doppler examination of the ductus venosus (DV) flow velocity waveforms (FVW) has been widely used in fetal surveillance and has shown that late changes in the pulsatility index (PI) of the DV-FVW might be a significant indicator for the decision of delivery in the management of FGR fetuses [4–7]. These evidences are based on the characteristic feature of DV; atrial and ventricular wall motions are transmitted by the blood flow of venous return, providing important information of fetal cardiac function [5–8].

We recently studied the DV-FVW from new aspects, using the time intervals of DV-FVW components in high risk fetuses [8–11]. In twin-twin transfusion syndrome (TTTS), recipients and donors showed characteristic changes in the time intervals of DV-FVW components, and laser therapy to coagulate the inter-twin anastomosis had significant impacts on these variables, thus suggesting that the time intervals of DV-FVW components might reflect hemodynamic changes caused by unbalanced blood volume between recipients and donors [9]. More recently, we have reported that the alterations of the time intervals of DV-FVW in severe FGR fetuses reflect the changes of the cardiac cycles in both ventricles, therefore suggesting pathological events caused by placental insufficiency [10].

The aims of this study were to evaluate the time intervals of DV-FVW and cardiac cycles, as well as DV-PI, in correlation with umbilical artery pH (UA-pH) at birth in growth-restricted fetuses complicated with placental insufficiency.

## Methods

### Participants

This study was retrospectively performed in Osaka City University Hospital and Osaka City General Hospital from April 2011 to January 2020. Data were retrieved from forty-one pregnant women complicated by FGR. Twenty-three of these cases were included in a previous study [10]. FGR was defined as an estimated fetal weight < − 2.0 SD of the local reference range with an elevated umbilical artery pulsatility index (UA-PI) > 95th percentile of the reference range [12, 13]. The last menstrual period was applied for the calculation of gestational age and the measurement of a crown–rump length at 9–11 weeks’ gestation was used for confirmation of gestational age. We retrospectively used the last examination for analysis, and the decision of delivery was made by non-reassuring fetal status. The exclusion criteria were multiple pregnancy, chromosomal abnormalities or structural anomalies.

### Measurements

**Fig. 1:**
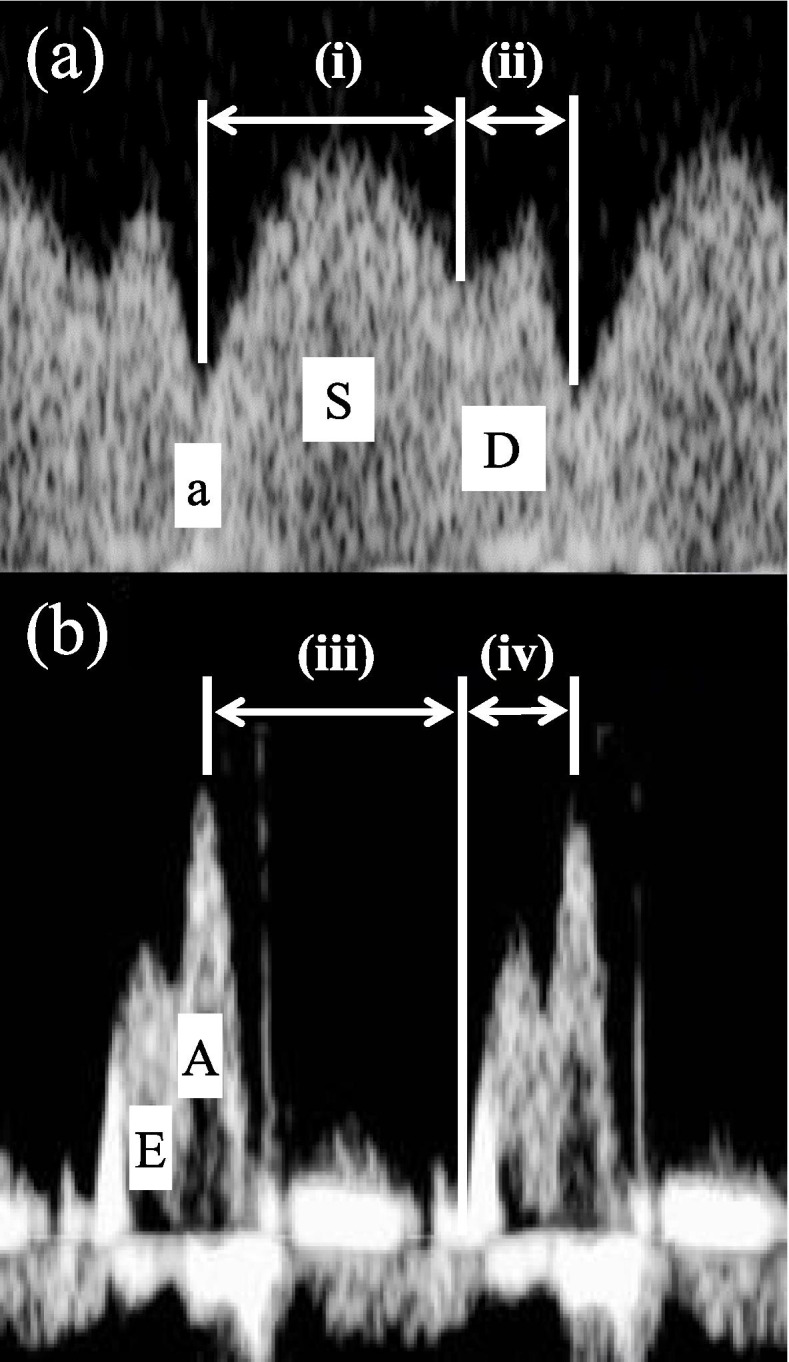
Doppler tracings of flow velocity waveforms (FVW) of ductus venosus (DV) (**a**) and ventricular inflow (**b**). (i) indicates time interval for S-wave of DV-FVW, measured from nadir of a-wave during atrial contraction to nadir between S-wave and D-wave; (ii) indicates time interval for D-wave of DV-FVW, measured from nadir between S-wave and D-wave to nadir of a-wave; (iii) indicates time interval from the second peak of ventricular inflow caused by atrial contraction (A-wave) to the opening of the tricuspid and mitral valves; (iv) indicates time interval from the opening of the tricuspid and mitral valves to the peak of the A-wave. (i)/(ii) expressed as DV-S/D. (iii)/(iv) was expressed as TV-S/D for tricuspid valve, and MV-S/D for mitral valve, respectively

The measurements were performed by T.S, D.T, N.Y, and Y.K. The methods of Doppler studies have already been mentioned in the literature [10]. Briefly, the angle between the ultrasound beam and the direction of blood flow was < 20° during the absence of fetal breathing movements. Either a midsagittal section or an oblique transverse section was used for the DV visualization and measurement. A four-chamber view was visualized to measure ventricular diastolic filling patterns. As shown in Fig. 1b, the time-interval measurements were taken for DV-FVW: (i), from the bottom of the a-wave during atrial contraction to the bottom between the S-wave and D-wave and: (ii), from the bottom between the S-wave and D-wave to the bottom of the a-wave (Fig. 1a). (i)/(ii) was defined as DV-S/D. The following time intervals of ventricular inflow through both the tricuspid valve (TV) and the mitral valve (MV) were obtained for assessment of the cardiac cycles: (iii), from the second peak of ventricular inflow caused by atrial contraction (A-wave) to the opening of the tricuspid and mitral valves and: (iv), from the opening of the tricuspid and mitral valves to the peak of the A-wave. (iii)/(iv) was defined as TV-S/D and MV-S/D, for the tricuspid valve and the mitral valve, respectively. We excluded the cases with poor signal quality from the study. The measurements were undertaken during the fetal heart rate was kept within the normal range of 120–160 bpm and differences among measurements in each fetus were < 5 bpm. The data obtained within 4 days before delivery were used for statistical analysis.

### Statistical analysis

We used the SPSS statistics version 20 for statistical analysis (SPSS Inc., Chicago, IL, USA). Formula was derived by regression analysis for the relationship with gestational age and reference ranges were calculated for the value of S/D [10]. We transformed the measurements into z-scores for comparison with reference ranges according to the following formula: z-score = (X_GA_ − M_GA_) /SD_GA_, where X_GA_ is the measured value at a known gestational age, M_GA_ is the mean value according to the reference equation and SD_GA_ is the standard deviation associated with the mean value at this gestational age [10]. We calculated correlations between parameters and UA-pH using the Pearson’s correlation coefficient and *P* < 0.05 was considered statistically significant.

### Ethical approval

All participants gave informed written consent and the study protocol was approved by the institutional review board of Osaka City University Graduate School of Medicine on 1 October 2020 (Approved Number: 2020–183).

## Results

### Maternal characteristics and neonatal outcomes

During the observational period, 10 cases out of the original 41 FGR cases were excluded; one case had a ventricular septal defect that was diagnosed postnatally, one showed fetal tachycardia, one resulted in fetal demise, and 7 cases were delivered with the decision of maternal indication such as hypertensive disorder in pregnancy. Twenty-one cases were included in previous study [10]. Of the 31 growth-restricted fetuses, measurements of DV-S/D and DV-PI were obtained from all cases, TV-S/D from 22 cases and MV-S/D in 23. All cases were delivered by cesarean section due to non-reassuring fetal status. No case was complicated with placental abruption. The mean of time intervals between the last Doppler examination and delivery was 1.1 day. Maternal characteristics and neonatal outcomes are shown in Table 1.

**Table 1:** Maternal characteristics and neonatal outcomes of this study

		GA (weeks) at examination:	
Maternal characteristics Neonatal outcomes	All (*n* = 31)	≤ 28 + 6 (*n* = 13)	> 28 + 6 (*n* = 18)
Age at delivery (years)	31.1 (±4.9)	31.8 (±4.8)	30.7 (±5.1)
Nulliparous/parous	22 / 9	11 / 2	11 / 7
Measurement before delivery (day)	1.1 (±1.4)	0.8 (±1.2)	1.3 (±1.5)
GA at delivery (weeks)	30.0 (±3.2)	27.2 (±1.4)	32.3 (±2.2)
Birth weight (g)	855 (±373)	523 (±105)	1058 (±332)
1-min Apgar score	5.9 (±2.0)	4.3 (±1.4)	6.9 (±1.5)
5-min Apgar score	7.5 (±1.6)	6.1 (±1.6)	8.3 (±1.0)
Umbilical artery pH	7.21 (±0.10)	7.19 (±0.13)	7.21 (±0.07)
Umbilical artery base excess	−4.7 (±3.9)	− 4.4 (±3.3)	−4.9 (±4.2)

Data are given as mean (±SD) or number

*GA* gestational age

### Correlations of each parameter with UA-pH

**Fig. 2:**
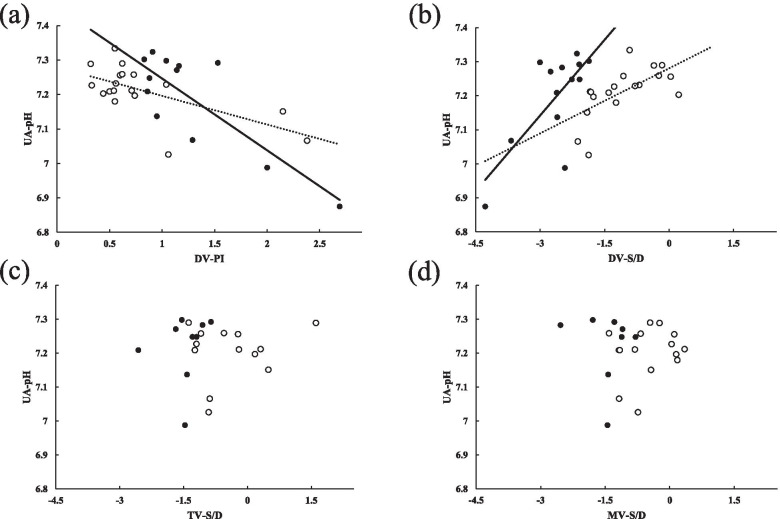
Correlation between the umbilical artery pH (UA-pH) and each parameter: **a** DV-PI, **b** z-score of DV-S/D, **C** z-score of TV-S/D, and **d** z-score of MV-S/D. The values of GA at exam ≤28 + 6 weeks, and GA at exam > 28 + 6 weeks were indicated as ● and 〇, respectively. Solid lines indicate the regression line for the value of ≤28 + 6 weeks cases and dotted lines indicate the regression line for the value of > 28 + 6 weeks cases. **a** solid line; y = − 0.2085x + 7.4548, dotted line; y = − 0.0835x + 7.2797, **b** solid line; y = 0.1481x + 7.586, dotted line; y = 0.0637x + 7.2807, **c** solid line; y = 0.0389x + 7.2758, dotted line; y = 0.0157x + 7.2101, **d** solid line; y = − 0.0208x + 7.1900, dotted line; y = 0.0297x + 7.2155

The data of results are summarized in Table 2. Both DV-PI and z-score of DV-S/D showed a significant correlation with UA-pH (*r* = − 0.677, *p* = < 0.001 and *r* = 0.489, *p* = 0.005 for DV-PI and z-score of DV-S/D, respectively) and more significances were observed in FGR ≤ 28 + 6 gestational weeks (*r* = − 0.819, *p* < 0.001 and *r* = 0.726, *p* = 0.005, for DV-PI and z-score of DV-S/D, respectively) than in FGR > 28 + 6 gestational weeks (*r* = − 0.634, *p* = 0.007 and *r* = 0.635, *p* = 0.020, for DV-PI and z-score of DV-S/D, respectively). On the other hand, TV-S/D and MV-S/D showed no significant correlation with UA-pH, although these z-scores indicated significant decreases compared with normal references (Table 2). Figure 2 shows the results of each variables.

**Table 2:** Correlations of each parameters of ductus venosus flow velocity waveforms (DV-FVW) measurements and right and left ventricular measurements in fetal growth restriction, overall and according to gestational age (GA) at examination (exam)

Parameters	All	*r*	*P*	GA at exam ≤ 28 + 6 weeks	*r*	*P*	GA at exam >28 + 6 weeks	*r*	*P*
DV-FVW	*n* = 31			*n* = 13			*n* = 18		
FHR (bpm)	141 (±9)			139 (±9)			143 (±7)		
DV-PI	0.987 (±0.58)	−0.677	< 0.001	1.243 (±0.53)	− 0.819	< 0.001	0.802 (±0.55)	−0.634	0.007
DV-S/D (ratio)	1.693 (±0.38)	0.428	0.016	1.425 (±0.23)	0.552	0.050	1.886 (±0.35)	0.518	0.028
DV-S/D (z-score)	−1.724 (±1.04)^*^	0.489	0.005	−2.637 (±0.65) ^*^	0.726	0.005	− 1.065 (±0.72) ^*^	0.635	0.020
Right ventricle	*n* = 22			*n* = 9			*n* = 13		
FHR (bpm)	142 (±7)			143 (±8)			141 (±6)		
TV-S/D (ratio)	2.218 (±0.45)	−0.017	0.940	2.035 (±0.26)	0.090	0.817	2.346 (±0.51)	−0.018	0.952
TV-S/D (z-score)	−0.826 (±0.87) ^*^	0.060	0.789	−1.453 (±0.45) ^*^	0.189	0.626	−0.392 (±0.83)	0.169	0.580
Left ventricle	*n* = 23			*n* = 9			*n* = 14		
FHR (bpm)	142 (±6)			142 (±7)			142 (±6)		
MV-S/D (ratio)	1.865 (±0.70)	−0.159	0.465	1.761 (±0.22)	−0.147	0.706	1.933 (±0.19)	−0.119	0.687
MV-S/D (z-score)	−0.825 (±0.70) ^*^	−0.006	0.979	−1.412 (±0.48) ^*^	−0.105	0.788	−0.448 (±0.54)	0.218	0.566

Date are given as mean (±SD) or number. Each z-score of FGR fetuses was compared with that of the control group [10] using the Pearson’s correlation coefficient

*bpm* beat per min, *DV* ductus venosus, *TV* tricuspid valve, *MV* mitral valve

^*^ significant difference compared with normal population [10]

## Discussion

This study firstly investigated the correlations of UA-pH at birth and the time interval parameter of DV-FVW and cardiac cycles, as well as DV-PI in FGR cases. DV-S/D and DV-PI showed significant correlations with UA-pH, especially in the FGR ≤ 28 + 6 gestational weeks. On the other hand, parameters of cardiac cycles such as TV-S/D and MV-S/D did not correlate with UA-pH, although both of them were significantly decreased when compared with normal reference ranges. These observations suggest that the pathological hemodynamic condition in severe FGR fetuses might be more sensitively reflected not only in pulsatility, but also in the time interval of DV-FVW, rather than in cardiac cycles.

Nakagawa et al. studied the time intervals of DV-FVW in normal fetuses and revealed their physiological properties in correlation with gestational changes of ventricular diastolic function [8]. More recently, Wada et al. showed a significant decrease of DV-S/D, TV-S/D and MV-S/D in FGR fetuses using the normal reference ranges and proposed the theory that functional hypovolemia and decreased ventricular pressure caused by pathological circulation due to placental insufficiency might extend the ventricular filling time (in other words, ventricular diastolic time), thus leading to decreased TV- and MV-S/D in FGR fetuses, and that these alterations are well reflected in DV-S/D [10]. This theory might be supported by reports of strikingly decreased DV-S/D in the donor of TTTS cases [9] and a case of anemia/hypovolemia caused by massive hemorrhage [11]. Regarding UA-pH at birth, however, correlations were observed in parameters of DV-FVW but not in cardiac ones. A possible explanation might be as follows; time intervals of DV-FVW might be affected by not only atrio-ventricular opening/closing duration but also by the complexity of cardiac compliance, contractility, vascular resistance, blood viscosity and blood volume [9–11, 14–17].

Based on the theory that cardiac wall motion generates a biphasic pattern with two peaks and two troughs of DV-FVW, several studies evaluated parameters using each velocity of DV-FVW in relation with perinatal outcomes and cardiac function [18, 19]. Picconi et al. assessed a Doppler index incorporating peak systolic waves and two periods of deceased velocity during atrial contraction and isovolumetric relaxation in severe premature FGR fetuses and their index was shown to be a useful parameter for a prediction of fetal outcomes [18]. Sanapo et al. also assessed the velocity ratios using the velocity of the trough between the systolic and diastolic wave (they named the trough as ‘v’- wave during end-systolic ventricular relaxation) in various diseases including FGR fetuses. They successfully showed correlations with cardiac function such as E/A ratios (E: peak velocity of early passive ventricular filling wave, A: peak velocity of late active ventricular filling wave) and myocardial performance index, although pulsatile index for veins of DV did not correlate with these cardiac indices in their study [19]. As such, analysis of DV-FVW from different angles will provide various insights for the evaluation of FGR pathophysiology.

One of the primary goals for severe FGR cases caused by placental insufficiency is antenatal prediction of UA-pH and delivery decision at an optimal timing [20–22]. For this purpose, continuous close monitoring is essential in the clinical practice. The limitation of this study is the paucity of sequential data analysis, including the data from days previous to the day of the delivery. In addition, we could not obtain complete measurements of every parameter in some cases. However, the strengths are that this is the first report which has investigated time interval parameters of DV-FVW and cardiac cycles in relation with UA-pH at birth. Moreover, our observation revealed that DV-S/D might be a predictive parameter of fetal UA-pH in placenta insufficiency.

## Conclusion

We showed that DV-S/D is a useful parameter in the management of FGR fetuses regarding the prediction of fetal acidemia. We believe that time interval analysis of DV-FVW might be a valuable parameter in the antenatal surveillance of fetuses at high risks. Longitudinal studies are further needed to monitor when the alteration of DV-S/D becomes apparent in the process of fetal deterioration in FGR cases with placental insufficiency.

## Supplementary Information

**Additional file 1.**

## Data Availability

All data related to this study are contained within the manuscript.

## Abbreviations

FGR: Fetal growth restriction
DV: Ductus venosus
FVW: Flow velocity waveform
UA: Umbilical artery
PI: Pulsatile index
TV: Tricuspid valve
MV: Mitral valve
S/D: Systolic / diastolic ratio
